# Volatile Metabolic Markers for Monitoring *Pectobacterium carotovorum* subsp. *carotovorum* Using Headspace Solid-Phase Microextraction Coupled with Gas Chromatography-Mass Spectrometry

**DOI:** 10.4014/jmb.2009.09028

**Published:** 2020-11-14

**Authors:** Ji-Su Yang, Hae-Won Lee, Hyeyeon Song, Ji-Hyoung Ha

**Affiliations:** Hygienic Safety and Analysis Center, World Institute of Kimchi, Gwangju 61755, Republic of Korea

**Keywords:** Cabbage, *Pectobacterium*, soft rot, solid-phase microextraction, volatile metabolic marker

## Abstract

Identifying the extracellular metabolites of microorganisms in fresh vegetables is industrially useful for assessing the quality of processed foods. *Pectobacterium carotovorum* subsp. *carotovorum* (PCC) is a plant pathogenic bacterium that causes soft rot disease in cabbages. This microbial species in plant tissues can emit specific volatile molecules with odors that are characteristic of the host cell tissues and PCC species. In this study, we used headspace solid-phase microextraction followed by gas chromatography coupled with mass spectrometry (HS-SPME-GC-MS) to identify volatile compounds (VCs) in PCC-inoculated cabbage at different storage temperatures. HS-SPME-GC-MS allowed for recognition of extracellular metabolites in PCC-infected cabbages by identifying specific volatile metabolic markers. We identified 4-ethyl-5-methylthiazole and 3-butenyl isothiocyanate as markers of fresh cabbages, whereas 2,3-butanediol and ethyl acetate were identified as markers of soft rot in PCC-infected cabbages. These analytical results demonstrate a suitable approach for establishing non-destructive plant pathogen-diagnosis techniques as alternatives to standard methods, within the framework of developing rapid and efficient analytical techniques for monitoring plant-borne bacterial pathogens. Moreover, our techniques could have promising applications in managing the freshness and quality control of cabbages.

## Introduction 

Postharvest decay occurs naturally along with changes in microbial communities and physicochemical properties during long-term storage of fresh produce. Simultaneously, numerous volatile substances are also released, the roles of which may be useful from various perspectives [1]. A previous study reported that volatile compounds (VCs) produced by fungi have biotechnological potential for controlling postharvest decay [2]. Another study demonstrated the application of VCs for controlling postharvest fruit diseases caused by *Muscodor albus* [3]. VCs generated by antagonist bacteria have also been suggested as effective for controlling postharvest decay caused by plant pathogens but have not been studied in detail [4, 5]. In contrast, several studies have demonstrated that plant pathogenic bacteria can alter the pattern of VCs emitted from fresh vegetable tissues [6, 7]. Interestingly, the biological characteristics reflected by emitted VCs can be considered as specific disease markers. Studies have also shown that many plant pathogens (*e.g.*, *Pectobacterium carotovorum* subsp. *carotovorum* (PCC), *P. carotovorum* subsp. *atrosepticum*, *Phytophthora infestans*, *Pythium ultimum*, *Botrytis cinerea*, and *Fusarium sambucinum*) generate volatile metabolic markers that enable identification of fruit and vegetable infections [7].

PCC is a gram-negative pathogenic bacterium that causes soft rot disease in vegetables and plants including cabbage, potato, onion, and radish during cultivation, transportation, and storage, resulting in considerable economic losses [8]. It exists in soil and on plant surfaces and may penetrate host cells through natural openings or wound sites [8]. PCC is a pectinolytic pathogen, producing several numbers of pectin- and cellulose-degrading enzymes that can catalyze the breakdown of pectin, the primary plant cell wall component [9]. After stably penetrating the plant tissues, PCC settles in the intercellular spaces, where it reproduces and causes disease [10]. Residual PCC can also emit VCs that may contain host cell tissues and PCC species [10]. According to Turner and Magan [11], the type of plant tissues and microbial species present can affect the pattern and amount of volatile molecules produced. Therefore, specific VCs generated in the presence of PCC may be useful as volatile markers to detect contaminated produce. In recent decades, various analytical instruments such as gas sensor arrays [12], metal-oxide gas sensors [13], electronic nose [14], gas chromatography-mass spectrometry [15], headspace solid-phase microextraction followed by gas chromatography coupled with mass spectrometry (HS-SPME-GC-MS)[16], and high-field asymmetric waveform ion mobility spectrometry [17] have been employed to analyze VCs for the early detection or monitoring of plant diseases, specifically VCs in vegetables other than cabbage including potatoes, tomatoes, and onions [13, 16].

Solid-phase microextraction (SPME) is a technique for experiment-friendly sample preparation that combines sample extraction and sample introduction in a single step while completely eliminating or minimizing the use of solvents [18]. It uses the fused-silica fiber coated with an appropriate stationary phase in SPME, which plays a key role in determining the efficiency of SPME [19]. SPME combined with headspace-involving nondestructive sample preparation has been widely used for food flavor, environmental, and chemical analyses [20]. Moreover, SPME has been used to identify various VCs along with GC/MS and to detect trace amounts of VCs in the headspace of cabbage kimchi samples [21].

To our knowledge, reports on the various VCs in cabbage (*Brassica rapa* subsp. *pekinensis*) contaminated with PCC are limited. Therefore, in this study, we used automated HS-SPME-GC–MS-based extracellular metabolomics as an analytical approach for identifying VCs related to PCC in cabbage. The study was conducted to determine whether HS-SPME-GC-MS could differentiate VCs emitted from cabbage samples under different conditions by measuring their headspace volatiles. We also characterized the VC profiles and determined the specific volatile metabolic markers of different sample groups (fresh cabbage without external infection symptoms and artificially infected cabbage showing external infection symptoms, at different storage temperatures) using GC-MS at the laboratory scale.

## Materials and Methods

### Bacterial Strains

PCC (strain PC1) originally isolated from cabbage (*B. rapa*, subsp. pekinensis) was kindly provided by the National Institute of Agricultural Sciences Rural Development Administration [22]. PCC was grown in lysogeny broth medium (1% tryptone, 0.5% yeast extract, 1% NaCl, pH 7.5; Difco Laboratories, USA) at 28°C for 20 h before use in subsequent experiments.

### Cabbage Sample Preparation and Microbiological Analysis

The cabbage ‘Choongwang’ cultivar was purchased from an agricultural wholesale market in Gwangju, Korea. Each head of cabbage was thoroughly washed for 30 min with running, slightly acidic electrolyzed water (SAEW; free available chlorine content 30 ppm) as a disinfectant. The bruised outer leaves were eliminated. Fresh, intact cabbage leaves (approximately 100 g) were collected and used to identify the VCs for HS-SPME-GC-MS analysis. Prior to inoculation, all cabbage leaves were sanitized by soaking in SAEW for 30 s each. For PCC inoculation, small, cross-shaped scars were made with a sterile knife on several cabbage leaves that were then spiked with 100 μl of PCC suspension [3.93 log_10_ colony-forming units (CFU)/ml]. Inoculations were performed in a sterilized biosafety cabinet. Each inoculated cabbage sample was stored temporarily at 10 ± 2°C for 30 min until they were dried completely to enable the PCC bacterial cells to attach evenly. For VC profiling, the SPME fiber was exposed to the headspace of cabbage samples stored for 7 days at 0°C, 10°C, 20°C, or 30°C. In addition, to quantify microbe-induced plant volatiles, the PCC suspension (3.93 log_10_ CFU/ml) was centrifuged at 6,000 ×*g* for 20 min, and the pellet was resuspended in phosphate-buffered saline (pH 7.4). The bacterial pellet (denoted as Bpellet) was defined as a control group and was stored for 7 days. Furthermore, VC profiling of Bpellet samples was performed under various temperature conditions, including 0°C, 10°C, 20°C, and 30°C. The moisture content was measured with an infrared moisture analyzer (MB45, Ohaus Corp., USA) after homogenizing four pieces of outer head leaves. The moisture content of the fresh cabbage measured in triplicate immediately before PCC inoculation was 93.23 ± 1.67%. For PCC quantification, each cabbage sample was placed in a sterile filter stomacher bag (Seward Limited, UK) with 90 ml of 1 g/l peptone water (PW; Oxoid, UK) and was evenly blended using a stomacher (Elmex SHII M; Japan) for 1 min. Next, 1 ml of the sample was serially diluted by 10-fold in 1 g/l PW. One milliliter of each dilution was plated on tryptic soy agar (TSA; Difco Laboratories), and the diluted samples were aseptically placed in a Petri dish. Approximately 20–25 ml of tryptic soy agar at 55°C was poured on the plates. The Petri dishes were incubated at 28°C for 20 h, and the PCC colonies appearing on the plates were counted using the standard plate count method and expressed as CFU/g. Additionally, we quantified the viable PCC cells in cabbage samples by quantitative reverse transcriptase PCR assays combined with an intercalating propidium monoazide dye [23]. PCC bacterial DNA was not detected in some cabbage samples, or PCC bacterial DNA was detected in no cabbage samples (data not shown).

### HS-SPME-GC-MS Analysis: Solid Phase Microextraction

HS-SPME was performed using a multipurpose autosampler (MPS 2; Gerstel, Germany). First, the cabbage samples (approximately 5 g) were placed in a headspace glass vial of 20 ml with a screw neck and incubated for 5 min at 60°C for saturation of VCs released from the samples in the vial headspace. After incubation, an SPME fiber comprising materials such as divinylbenzene/carboxen/polydimethylsiloxane (50/30 μm; Supleco Inc., USA) was inserted into the headspace of the vial, and the VCs saturated in the vial were absorbed by the SPME fiber for 20 min at 60°C with shaking at 300 rpm. After absorption, the SPME fiber was inserted into the inlet port of the gas chromatograph-mass spectrometer (GC-MS) set to splitless mode at 250°C. After insertion, VCs from the SPME fiber were desorbed over 1 min in the inlet port of the GC-MS. A bake-out process of the SPME fiber was performed for 10 min at 200°C after GC-MS analysis.

### HS-SPME-GC-MS Analysis: Gas Chromatography-Mass Spectrometry

The VCs released from samples by the SPME fiber were analyzed using a GC system (Agilent 7890A; Agilent Technologies, USA) coupled with a MS (5977B; Agilent). This GC-MS was equipped with a DB-WAX capillary column (60 m × 0.25 mm × 0.25 μm film thickness; Agilent). Helium (99.999% or more) was used as the carrier gas, and the flow rate was set to 1 ml/min. The temperature of the inlet set to splitless mode was 250°C. The ramp condition of the oven was as follows: held at 40°C for 3 min, increased to 150°C at 2°C/min, held at that temperature for 10 min, increased to 200°C at 4°C/min, and held at that temperature for 10 min. The temperatures of the thermal AUX and ion source of the mass spectrometer were set to 280°C and 230°C, respectively. The mass scan range was set to 35–400 m/z, and the electron ionization energy was set to 70 eV. The peaks on the total ion chromatogram were detected and identified as VCs using MassHunter qualitative analysis software (version B.07.00; AgilentA) with the WILEY10N library.

### Statistical Analysis 

To compare the experimental groups, principal component analysis (PCA) and partial least squares-discriminant analyses (PLS-DA) were performed based on normalization using MetaboAnalyst 4.0 [24]. The degree of difference in VCs between the experimental groups was identified by variable importance in projection (VIP) scores estimated as a weighted sum of squares of the PLS loadings, considered the amount of explained y-variation for each dimension. Three experimental replications were performed for each cabbage sample.

## Results and Discussion

### Growth Profile of PCC on Cabbage

**Fig. 1:**
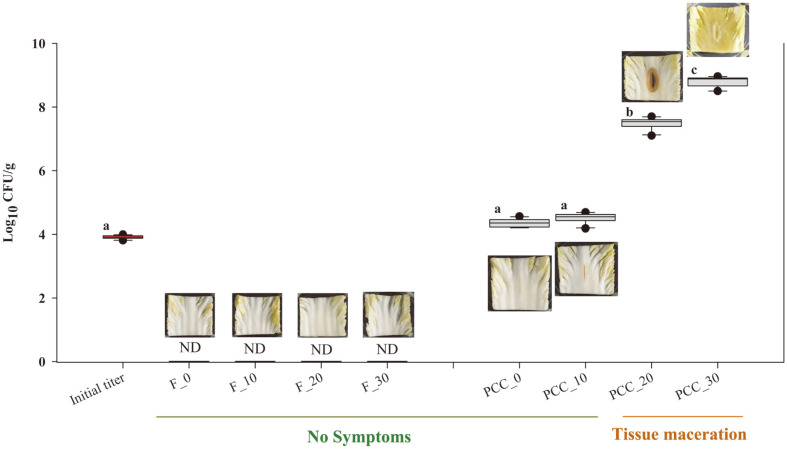
Comparison of *Pectobacterium carotovorum* subsp. *carotovorum* (PCC) bacterial counts and soft rot development between fresh cabbages and PCC-inoculated cabbages under different storage temperatures after 3 days. Fresh cabbage samples stored at 0°C, 10°C, 20°C, and 30°C are denoted as F0, F10, F20, and F30, respectively. PCC-inoculated cabbages stored at 0°C, 10°C, 20°C, and 30°C are denoted as PCC0, PCC10, PCC20, and PCC30, respectively.

We investigated the growth properties of PCC on cabbage samples under different temperature conditions and cabbage tissue maceration based on soft rot on fresh-cut cabbage that had been sterilized before PCC inoculation to eliminate background microbial populations. The quantitative data and maceration symptoms are shown in Fig. 1. For all fresh cabbage samples, no maceration symptoms were observed for 3 days, whereas PCC-inoculated cabbage samples, except those stored at less than 10°C, showed obvious maceration symptoms. The PCC growth pattern also changed, and the bacterial population increased with increasing storage temperature. The bacterial counts showed no significant differences in PCC-inoculated cabbage samples stored at 0°C and 10°C (~4.31 and 4.49 log_10_ CFU, respectively) compared to the initial number of bacteria inoculated on the cabbage samples (3.93 log_10_ CFU). However, the mean values of PCC on the cabbage samples increased to 7.58 log_10_ CFU (stored at 20°C) and 8.97 log_10_ CFU (stored at 30°C), respectively. Similar effects of temperature on soft rot were earlier reported by Bhat *et al*. [25], who showed that soft rot can occur at temperatures as low as 16°C to those above 35°C. Furthermore, Agrios [26] demonstrated that high temperature is closely related to PCC proliferation. High temperatures generate optimal maceration conditions as oxygen in the tissue is rapidly replaced with a high carbon dioxide content. In accordance with these previous results, soft rot infection is likely to occur at inappropriate storage temperatures above 10°C.

### HS-SPME-GC-MS Analysis for Soft Rot Disease in Cabbage Samples

For profiling of the VCs, the SPME fiber was exposed to the headspace of cabbage samples stored for one week at different temperatures. Various VCs emitted by two types of sample groups (fresh and artificially PCC-infected cabbage samples) stored at different temperatures were detected by SPME-GC-MS analysis (Table S1). The increase in the specific concentrations of VCs with PCC inoculation was related to tissue maceration in cabbage compared to that in fresh cabbage samples without PCC inoculation. Indeed, the release pattern of VCs was similar to the pattern of soft rot symptoms on cabbage tissue. Univariate and multivariate analyses clearly revealed these results.

### PCA of HS-SPME-GC-MS Analysis

**Fig. 2:**
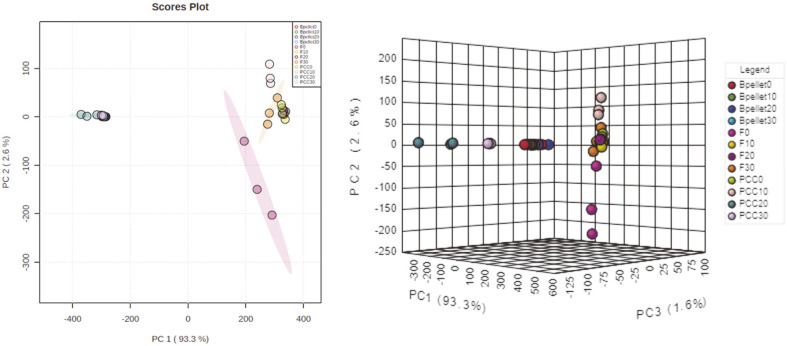
Principal component analysis (PCA) score plot of fresh and *Pectobacterium carotovorum* subsp. *carotovorum* (PCC)-infected cabbages. (**A**) Three-class discrimination using the headspace solid-phase microextraction followed by gas chromatography coupled with mass spectrometry (HS-SPME-GC-MS) analysis. (**B**) Fresh cabbage samples stored at 0°C, 10°C, 20°C, and 30°C are denoted as F0, F10, F20, and F30, respectively. PCC-inoculated cabbages stored at 0°C, 10°C, 20°C, and 30°C are denoted as PCC0, PCC10, PCC20, and PCC30, respectively. Control is denoted as Bpellet.

For volatile metabolite profiling, the SPME fiber was exposed to the headspace of passive samples stored for 7 days at different temperatures. All samples were subjected to PCA based on the profile of VCs emitted from the cabbage samples. Classification of passive samples based on their VC profiles by HS-SPME is shown in Figs. 2A and 2B. Principal component one (pc1) and principal component two (pc2) accounted for 93.3% and ca. 2.6% of the total data variability respectively (Fig. 2A). The VC information of all samples was covered by pc1 and pc2. These results indicate that our pc1 and pc2 analysis can be used to identify differences among all cabbage samples. Furthermore, PCC bacterial samples (Bpellet), fresh cabbages, and PCC-inoculated cabbages were divided into three regions of the PCA score plot that could be differentiated clearly. However, PCC-inoculated cabbage samples stored at 0°C and 10°C were similar to the fresh cabbage samples. These results indicate that PCC activation was insufficient to generate VCs because the contaminated cabbage tissue was stored below 10°C. The thermodependency of the soft rot disease occurrence and scale of the damage has been reported previously [27], demonstrating that active multiplication of PCC at the infection site followed by the production of numerous extracellular enzymes are prerequisites for disease development. Furthermore, PCC causes soft rot in the storage warehouse when temperatures are above 25°C [28] and is more often isolated from soft rot-infected tissues under high temperatures [29]. Interestingly, our results indirectly demonstrate that a suitable storage temperature required to maintain cabbage quality is lower than 10°C. For the 3D score plot, the first three PCs accounted for 97.5% of the total data variability among the Bpellets, fresh cabbages, and PCC-inoculated cabbages (Fig. 2B). The obtained PCs clearly distinguished the experimental data on a hyperplane that differentiated the fresh cabbages and PCC-inoculated cabbages.

### Correlation of HS-SPME-GC-MS Analysis

**Fig. 3:**
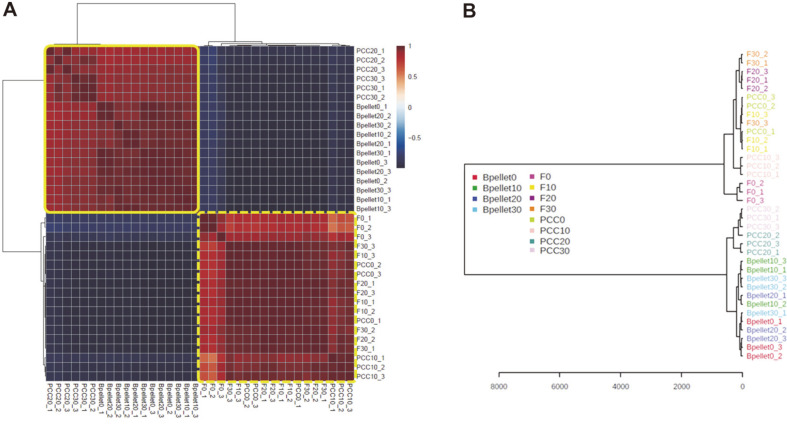
Correlation analysis clustering with Pearson’s correlation matrix heatmap (**A**) and the dendrogram (**B**) of different cabbage samples. Fresh cabbage samples stored at 0°C, 10°C, 20°C, and 30°C are denoted as F0, F10, F20, and F30, respectively. PCC-inoculated cabbages stored at 0°C, 10°C, 20°C, and 30°C are denoted as PCC0, PCC10, PCC20, and PCC30, respectively. Bpellet, the bacterial suspension pellet, was stored at 30°C as the control.

A correlation matrix heatmap was visualized using color-coded correlation matrices with a color gradient ranging from blue to red (Fig. 3A). In this representation, blue indicated a high negative correlation, whereas red represented a high positive correlation. Different cabbage groups identified two distinct clusters formed on the heatmap through the Pearson’s correlation approach. The upper left cluster comprised PCC-infected samples involving soft rot, whereas the bottom right cluster was mostly related to fresh cabbage samples, *i.e.*, some samples in this group were infected. Fig. 3B shows the individual PCA dendrograms obtained based on VC profile analysis for 7 days using passive sampling methods. As shown in Fig. 3A, significant differences were observed in the VCs between fresh and PCC-inoculated cabbage samples, except for the PCC-inoculated samples stored at 0°C and 10°C. The classification of fresh cabbage samples stored at 0°C, 10°C, 20°C, and 30°C and that of PCC-inoculated samples stored at 0°C and 10°C, based on the generated VCs, was similar. For profiling VCs from Bpellet under various temperatures such as 0°C, 10°C, 20°C, and 30°C, clear grouping with similarities was observed in the PCA dendrograms for PCC bacterial cells stored at 0°C, 10°C, 20°C, and 30°C (Fig. 3). The findings agree with those of previous studies on the effects of PCC on pectate lyase activity, which is involved in the maceration and soft rotting of plant tissue [30]. The study reported the thermodependency of pectate lyase activity, in addition to a significant association between the influence of temperature on pectate lyase activity and soft rot. Remarkably, the groups of PCC-inoculated samples stored at 20°C and 30°C and the remaining groups (fresh cabbage samples stored at 0°C, 10°C, 20°C, and 30°C) revealed completely contrasting behaviors. These results are consistent with those obtained from the PCA dendrograms (Fig. 3B). In the PCA dendrogram, nine color change profiles (Bpellet and two types of samples at four temperatures) were clustered in the order of their similarities. Most samples were closely gathered according to PCC infection, indicating that PCC infection could be correctly distinguished by different characteristics of VCs, as mentioned above. In addition, a clear grouping with similarities was observed in the PCA dendrograms for fresh cabbage samples stored at 0°C, 10°C, 20°C, and 30°C and for PCC-inoculated samples stored at 0°C and 10°C.

### HS-SPME-GC-MS Analysis of VCs in Cabbage Samples

**Fig. 4:**
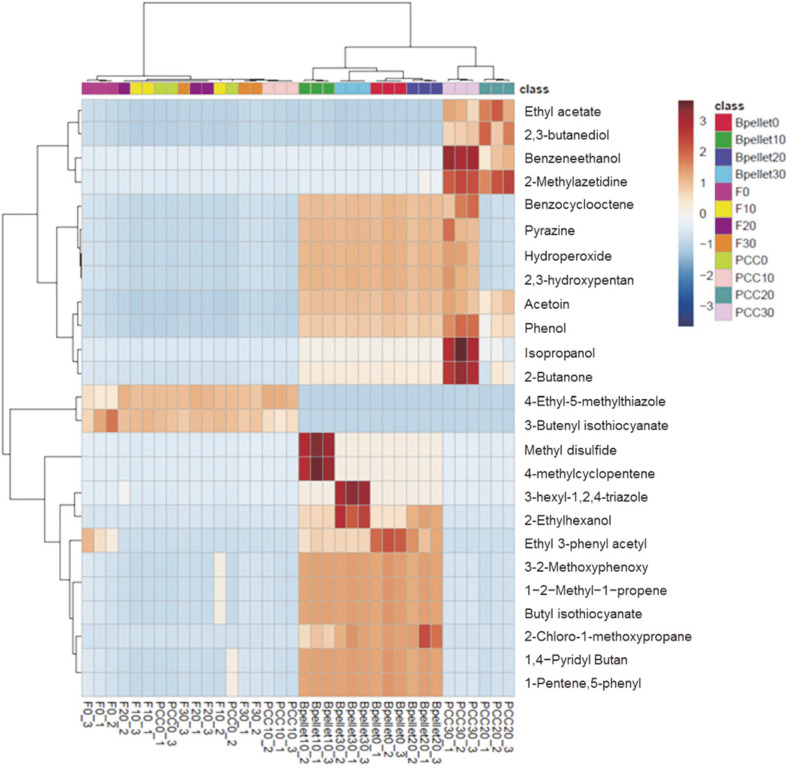
Clustering heatmap of the concentration of volatile compounds in cabbage samples at different storage temperatures. Fresh cabbage samples stored at 0°C, 10°C, 20°C, and 30°C are denoted as F0, F10, F20, and F30, respectively. PCC-inoculated cabbages stored at 0°C, 10°C, 20°C, and 30°C are denoted as PCC0, PCC10, PCC20, and PCC30, respectively. Bpellet, the bacterial suspension pellet, was stored at 30°C as the control.

The main VCs identified in the stored cabbage samples are listed in Table S1. The difference in the VCs of the two groups of cabbage samples at each storage temperature was analyzed using a clustering heatmap and comparable VCs, selected by a *t*-test (*p* < 0.01) (Fig. 4). As shown in Fig. 4, 2,3-butanediol, ethyl acetate, benzeneethanol, and 2-methylazetidine were the characteristic VCs in PCC-infected cabbages samples stored at 20°C and 30°C, mostly elicited by the bacterial soft rot disease symptoms. In contrast, 4-ethyl-5-methylthiazole and 3-butenyl isothiocyanate were emitted by fresh cabbage samples regardless of the storage temperatures and PCC-inoculated cabbages samples at 0°C and 10°C. PCC-inoculated cabbage samples stored at 0°C and 10°C for 7 days showed no soft rot symptoms, indicating that PCC cannot grow at temperatures below 10°C. Furthermore, Bpellet groups showed different patterns than the PCC-infected cabbage samples stored at 20°C and 30°C. This result suggested that VCs obtained from infected tissues were microbe-induced plant volatiles.

### PLS-DA of VCs

**Fig. 5:**
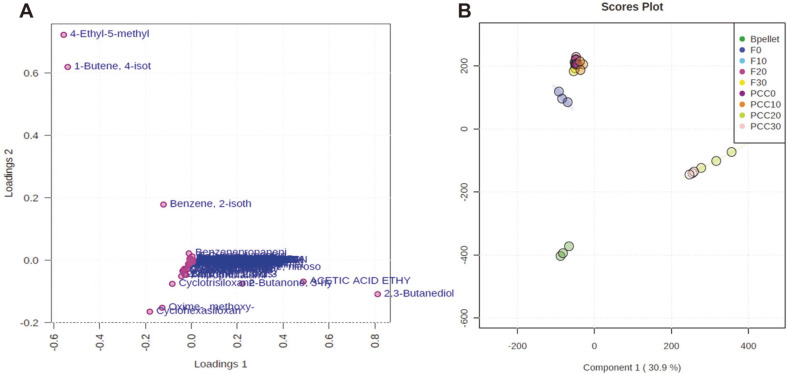
Partial least squares-discriminant analysis (PLS-DA) of headspace solid-phase microextraction followed by gas chromatography coupled with mass spectrometry (HS-SPME-GC-MS) analysis for each cabbage sample at different storage temperatures. Loading plot of PLS-DA (**A**) and PLS-DA scores scatter plot (**B**). Fresh cabbage samples stored at 0°C, 10°C, 20°C, and 30°C are denoted as F0, F10, F20, and F30, respectively. PCC-inoculated cabbages stored at 0°C, 10°C, 20°C, and 30°C are denoted as PCC0, PCC10, PCC20, and PCC30, respectively. Bpellet, the bacterial suspension pellet, was stored at 30°C as the control.

The VCs distributed on the loading plot map are shown in Fig. 5A. Separation of different groups of VCs analyzed from each cabbage sample during storage at different temperatures was represented by components 1 and 2. As shown in the PLS-DA scores scatter plot (Fig. 5B), PCC-infected cabbage samples were distributed in quadrant I. By combining the loading plot map and PLS-DA scores scatter plot map, 2,3-butanediol and ethyl acetate were identified as the major types of VCs in PCC-infected cabbage samples. Furthermore, 4-ethyl-5-methylthiazole and 3-butenyl isothiocyanate were the major types of VCs in fresh cabbages and PCC-inoculated cabbages without soft rot symptoms (stored at 0°C and 10°C).

### Identification of Metabolite Biomarkers

**Fig. 6:**
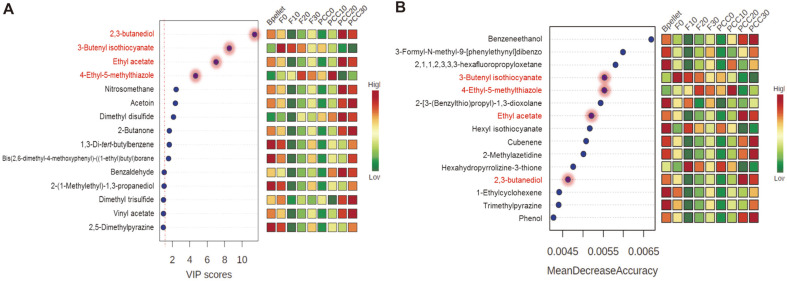
(**A**) Variable importance in projection (VIP) scores showing major volatile metabolites that are discriminatory and (**B**) random forest (RF) analysis showing the top 15 volatile metabolites responsible for classification as mean decreased accuracy values. Metabolite names provided in red font are shared between both analyses. Fresh cabbage samples stored at 0°C, 10°C, 20°C, and 30°C are denoted as F0, F10, F20, and F30, respectively. PCC-inoculated cabbages stored at 0°C, 10°C, 20°C, and 30°C are denoted as PCC0, PCC10, PCC20, and PCC30, respectively. Bpellet, the bacterial suspension pellet, was stored at 30°C as the control.

This study focused on detecting soft rot in cabbages by identifying volatile metabolic marker compounds for the disease using HS-SPME combined with GC-MS analysis. In recent years, the random forest (RF), a machine learning method, has been extensively used in the field of metabolomics for biomarker discovery [31, 32]. Additionally, it has been shown that the important features identified by PLS-DA through a typical multivariate analysis, such as VIP values, are effective for determining potential biomarkers [32]. Based on the PLS-DA model, we further analyzed the VIP values to determine the metabolites with VIP scores greater than 1, which represented important differential VCs. The results of volatile biomarker analysis indicated that 2,3-butanediol, 3-butenyl isothiocyanate, ethyl acetate, and 4-ethyl-5-methylthiazole were important metabolites for defining the PLS-DA model that distinguished the PCC-infected and non-infected cabbage samples (Fig. 6A). Using RF analysis, the features were ranked by a measure of permutation importance of the predictor’s classification of individual samples based on the mean decrease in accuracy. As shown in Fig. 6A, 2,3-butanediol, 3-butenyl isothiocyanate, ethyl acetate, and 4-ethyl-5-methylthiazole were the top four metabolites with overlap in both VIP and RF analysis. A previous study reported that metabolic profiles showed the concomitant appearance of a compound identified as 2,3-butanediol during PCC infection [33]. Butanediol, which accumulated during the symptomatic phase of the disease, is well known as a general metabolite found in rotted tissues of host plants. Butanediol is secreted in high concentrations by various *Pectobacterium* species during soft rot infection. Furthermore, butanediol has been identified as a signaling molecule in plant-bacterium interactions and, notably, enables induction of the plant’s systemic resistance. There have been many reports related to 2,3-butanediol as a volatile marker, indicating the presence of a plant pathogenic microorganism [34, 35]. Combined with the KEGG database, metabolic pathways of *P. carotovorum* subsp. *carotovorum* strain PCC21 (isolated from *B. rapa* L. ssp. *pekinensis*) were found to be associated with butanoate metabolism, which contributes to a rotting scent (Fig. S1). Marquez-Villavicencio *et al*. [36] reported that the 3-hydroxy-2-butanone pathway is required for *P. carotovorum* pathogenesis, and 2,3-butanediol plays an important role in volatile products. According to Kanchiswamy *et al*.[37], butanoate has been reported as a precursor for VC production or can enter VC metabolic pathways, as confirmed in the main pathway maps of butanoate metabolism by PCC21 (Fig. S1). Ethyl acetate is the most common ester and a general metabolite with a fruity smelling liquid found in fruits [38]. However, a high concentration of acetic acid may emit a VC having a strong, acetone-like odor. These volatile attributes enable masking of any positive feature associated with fruits and vegetables. Levey [39] demonstrated that general VCs include hydrocarbon alcohols and esters, such as ethanol and ethyl acetate, found in rotten fruits. In the VC profiles in the headspace of cabbage samples in our study, both 2,3-butanediol and ethyl acetate were specific volatile metabolic compounds. Notably, two major metabolites were observed in the groups of PCC-infected cabbages. These important VCs can be considered as specific metabolic biomarkers and may be used to discriminate cabbage conditions and the fresh status of cabbage from unknown sample groups.

## Conclusion

The HS-SPME-GC-MS technique allowed recognition of freshness in PCC-infected cabbages through identification of specific volatile metabolic markers. 4-Ethyl-5-methylthiazole and 3-butenyl isothiocyanate were markers specific to fresh cabbage, whereas 2,3-butanediol and ethyl acetate were specific to soft rot in cabbages infected with PCC. These analytical results demonstrate a suitable approach for realizing alternative non-destructive diagnostic techniques for plant pathogens, compared to standard methods within the framework of developing rapid and efficient analytical techniques for detecting plant bacterial pathogens. The technique also provides a promising alternative for freshness management and quality control in cabbages. However, more accurate and specific studies on metabolites are necessary to understand the detailed metabolic profiles of VCs. Moreover, identifying the VCs by the HS-SPME-GC-MS technique in real sample-scale experiments requires further anlaysis.

## Supplemental Material

Supplementary data for this paper are available on-line only at http://jmb.or.kr.
