# Supplementary material for: Volatile Metabolic Markers for Monitoring Pectobacterium carotovorum subsp. carotovorum Using Headspace Solid-Phase Microextraction Coupled with Gas Chromatography-Mass Spectrometry
Source: J Microbiol Biotechnol. 2020 Nov 14;31(1):70–8. doi: 10.4014/jmb.2009.09028 (PMC9705696; doi:10.4014/jmb.2009.09028)
Supplement: Supplementary file 1 [file jmb-31-1-70-supple.pdf]

J. Microbiol. Biotechnol.
https://doi.org/10.4014/jmb.2009.09028

Supplementary Figure S1.

Fig. S1. The major pathway maps of differential metabolites in butanoate metabolism by *Pectobacterium carotovorum* subsp. *carotovorum*.

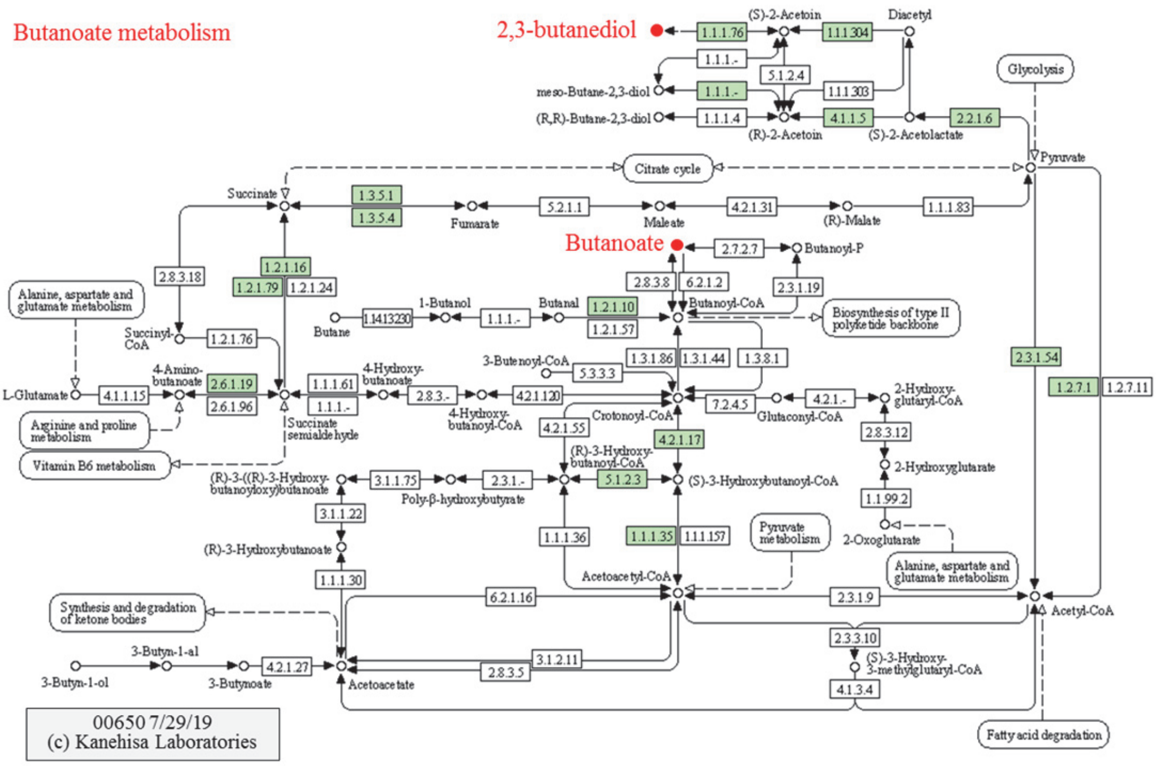

Supplementary Table S1.

No.	Volatile compounds	Formula	Peak area (×10^3)								
			PCC pellet	F0	F10	F20	F30	PCC0	PCC10	PCC20	PCC30
**1**	Allyl Isothiocyanate	C4H5NS	-	-	33	-	-	-	-	-	-
**2**	Palmitic acid	C16H32O2	100	-	-	-	-	-	-	-	-
**3**	Octadecanoic acid	C18H36O2	-	290	-	-	-	-	-	-	-
**4**	1,1-Dimethylhydrazine	C2H8N2	-	-	-	-	-	-	-	45	-
**5**	Benzeneethanol	C8H10O	-	-	-	-	28	-	25	316	694
**6**	Acetic acid	C2H4O2	-	-	-	-	-	-	-	174	72
**7**	Hexanal	C6H12O	-	10	-	23	32	-	34	52	67
**8**	Isopropyl alcohol	C3H8O	-	-	-	-	-	-	-	12	63
**9**	Acetaldehyde	C2H4O	-	-	-	-	-	-	-	11	-
**10**	Isopropylamine	C3H9N	-	-	-	-	-	-	33	-	-
**11**	*tert*-Butyl hydroperoxide	C4H10O2	-	-	-	-	-	-	-	-	53
**12**	2-Butanone	C4H8O	-	-	-	-	-	-	-	832	2592
**13**	Methyl acetate	C3H6O2	-	-	-	-	-	-	-	170	89
**14**	Methyl benzoate	C8H8O2	42	-	-	-	-	-	-	-	-
**15**	2,2-Dimethylvinyl cyclopropane	C7H12	-	-	13	-	-	-	-	-	-
**16**	Benzaldehyde	C7H6O	-	-	-	-	-	-	348	1456	1063
**17**	1,3-Di-*tert*-butylbenzene	C14H22	306	-	-	-	-	-	-	-	-
**18**	2-*O*-Methylstenosporic acid	C24H30O7	16	-	-	-	-	-	-	-	-
**19**	2-(1,1,2,3,3,3-Hexafluoropropyl)oxetane	C6H6F6O	-	-	-	16	11	-	27	-	22
**20**	2-Methyl-1-pentanol	C6H14O	11	-	-	-	-	-	-	-	-
**21**	Vinyl propionate	C5H8O2	-	-	-	26	-	-	-	18	-
**22**	Ethyl 2-butenoate	C6H10O2	-	-	-	-	-	-	-	27	-
**23**	Geraniol	C10H18O	-	-	-	-	-	-	31	46	81
**24**	Butyric anhydride	C8H14O3	-	-	20	16	30	-	-	-	-
**25**	(*E*)-*trans*-2,3-Epoxynon-7-enal	C9H14O2	-	-	-	-	21	-	-	-	-
**26**	Methaneselenoic acid, *O*-(1,1-diethylpropyl) ester	C8H16OSe	-	-	-	-	-	-	-	38	-
**27**	1-Phenyl-1,2-propanediol	C9H12O2	-	-	-	-	-	-	-	-	113
**28**	5,6,7,8,9,10-Hexahydrobenzocyclooctene	C12H16	-	-	-	-	-	-	-	-	16
**29**	Vinyl acetate	C4H6O2	-	-	-	-	-	-	-	1126	1149
**30**	*N*-[(Methylsulfonyl)oxy)]-*N*',*N*''-diphenyl-phosphoric triamide	C13H16N3O4PS	-	-	25	-	-	-	-	37	29
**31**	Toluene	C7H8	174	-	-	-	-	-	-	-	-
**32**	Cyclohexanone	C6H10O	22	-	-	-	-	-	-	-	-
**33**	Phenol	C6H6O	-	-	-	-	-	-	-	232	344
**34**	Pentane	C5H12	-	-	-	-	-	-	-	168	91
**35**	1-Methyl-4-[[3-methyl-2-[[4-methyl-3-(1-methylethyl)-2-pentenyl]ox	C21H32O3S	-	-	-	11	-	-	-	-	-
**36**	Isobutyl acetate	C6H12O2	-	-	-	-	-	-	-	43	-
**37**	6-Methyl-5-heptene-2-one	C8H14O	-	-	-	-	-	-	-	11	-
**38**	1-Hexanol	C6H14O	-	-	-	-	-	-	-	32	56
**39**	Heptanal	C7H14O	-	-	-	-	-	-	19	-	-
**40**	(*R*)-Citronellol	C10H20O	-	-	-	-	-	-	-	47	105
**41**	2-Undecanone	C11H22O	-	-	-	-	-	-	-	33	79
**42**	1-Decanol	C10H22O	29	-	-	-	-	-	-	28	30
**43**	Decanal	C10H20O	-	-	22	24	14	20	40	-	11
**44**	Octyl formate	C9H18O2	-	-	-	-	31	-	93	44	54
**45**	Tetramethylpyrazine	C8H12N2	-	-	-	-	-	-	-	-	23
**46**	3-Cyclohexyl-1-propanol	C9H18O	-	-	-	-	10	-	-	-	-
**47**	1-Dodecanol	C12H26O	106	-	-	-	-	-	-	26	23
**48**	5,6-Dimethyl-5-hepten-1-yne	C9H14	-	-	-	-	26	-	-	-	-
**49**	1-(4-Pentynyl)-pyrano[3,4-*b*]indol-3(9*H*)-one	C16H13NO2	11	14	154	-	-	-	-	-	-
**50**	1-Oxide-3-[(4-methylphenyl)amino]-4*H*-1-benzothiopyran-4-one	C16H13NO2S	65	78	69	93	152	55	94	133	129
**51**	2,4-Dichlorophenol	C6H4Cl2O	-	-	-	-	-	-	-	-	39
**52**	2,5-Dimethylpyrazine	C6H8N2	180	-	-	-	-	-	-	-	-
**53**	Isoamyl acetate	C7H14O2	-	-	-	-	-	-	-	118	109
**54**	Nonanal	C9H18O	-	11	15	25	20	28	46	15	12
**55**	2-[3-(Benzylthio)propyl)-1,3-dioxolane	C13H18O2S	-	-	19	-	11	-	-	-	-
**56**	*trans*-3-Methoxy-5-(4-methoxyphenyl)-1,2,4-trioxolane	C10H12O5	-	-	-	-	-	-	-	-	17
**57**	β-Phenethyl isothiocyanate	C9H9NS	-	769	9405	4988	4428	9030	4935	155	49
**58**	4-Nitrophthalamide	C8H7N3O4	94	116	77	58	77	68	151	156	127
**59**	Dimethyl 2,3-dimethylbut-2-enedioate	C8H12O4	-	23	-	-	-	-	-	-	-
**60**	3,3,5-Trimethyl-1-hexen	C9H18	-	15	-	-	-	-	-	-	-
**61**	*N*-(2-Furylmethyl)-*N*-methyl-1-phenyl-2-propanamine	C15H19NO	-	-	21	-	-	-	31	-	-
**62**	(*E*)-3,7-Dimethyl-2,6-octadienal	C10H16O	-	-	-	-	-	-	28	-	45
**63**	Ethyl acetate	C4H8O2	-	-	-	-	-	-	-	10749	6419
**64**	3a,4,7,7a-Tetrahydro-4,7-ethano-1*H*-isoindole-1,3(2*H*)-dione	C10H11NO2	-	18	-	20	-	21	14	-	-
**65**	1-Nonanol	C9H20O	-	-	-	74	37	-	103	-	23
**66**	3-Nonen-2-one	C9H16O	-	-	-	-	18	-	32	58	73
**67**	1-Ethylcyclohexene	C8H14	-	44	-	-	-	-	-	-	-
**68**	Trimethylpyrazine	C7H10N2	-	-	-	-	-	-	-	-	25
**69**	3-Propoxy-1-propene	C6H12O	-	-	-	-	-	-	10	-	-
**70**	β-Ionone	C13H20O	-	-	-	67	-	-	-	-	-
**71**	α-Longipinene	C15H24	-	-	-	-	-	-	-	-	22
**72**	2-Methylpentadecane	C16H34	-	-	-	-	-	-	-	12	-
**73**	(*Z*)-2-Penten-1-ol	C5H10O	-	-	-	-	-	-	10	-	-
**74**	1-Ethyl-1-methylcyclopentan	C8H16	-	10	-	-	-	-	-	-	-
**75**	3,6-Heptanedione	C7H12O2	-	-	-	-	-	-	-	-	17
**76**	1,4,7,10,13,16-Hexaoxacyclooctadecane	C12H24O6	-	-	-	15	-	-	-	-	-
**77**	α-Bergamotene	C15H24	-	-	-	-	-	-	-	-	87
**78**	2,4-Nonadienal	C9H14O	-	-	-	12	-	-	-	-	-
**79**	3,6,9,12,15-Pentaoxanonadecan-1-ol	C14H30O6	-	-	-	11	10	-	-	-	-
**80**	3-Octen-1-ol	C8H16O	-	-	-	11	10	-	-	-	-
**81**	(*E*)-β-Famesene	C15H24	-	-	-	-	-	-	-	-	62
**82**	(S,S)-2,3-Butanediol	C4H10O2	-	-	-	-	-	-	-	18311	10211
**83**	3-Methylhexanal	C7H14O	25	-	-	-	12	-	-	-	-

**84**	*cis*-Hexahydro-1,3-benzodioxol-2-one	C7H10O3	-	-	-	17	-	-	18	17	14
**85**	4,6-Dimethyl-2-heptanon	C9H18O	14	-	-	-	-	-	-	-	-
**86**	2-Methylazetidine	C4H9N	165	-	-	-	-	-	-	773	638
**87**	(*Z*)-2-Cyano-2-butene	C5H7N	-	-	74	83	80	-	-	-	-
**88**	*N*-Vinylpyrazole	C5H6N2	16	-	-	-	-	-	-	-	-
**89**	3-Cyclopropyl-1-butyne	C7H10	-	44	-	27	22	-	36	36	-
**90**	(*E*)-4-Hexen-3-ol	C6H12O	-	-	-	-	-	-	-	18	-
**91**	2-Hydroxy-3-pentanone	C5H10O2	-	-	-	-	-	-	-	-	59
**92**	6-Azabicyclo[3,2,0]heptan-7-one	C6H9NO	-	48	54	-	45	60	82	-	-
**93**	Butylisothiocyanate	C5H9NS	-	-	13	-	-	-	-	-	-
**94**	4,5-Dimethyl-1,3-dioxolan-2-one	C5H8O3	-	-	-	-	-	-	-	-	15
**95**	2-(1-Methylethyl)-1,3-propanediol	C6H14O2	291	-	-	-	-	-	-	-	-
**96**	Bis(*S*-Methyl)dithiocarbonate	C3H6OS2	-	-	-	-	-	-	-	17	28
**97**	(*Z,E*)-3,5-Octadien-2-one	C8H12O	-	26	16	26	39	17	26	-	34
**98**	*cis*-Oct-5-en-2-one	C8H14O	-	-	-	-	12	-	-	-	-
**99**	Tetrahydro-2-thiopheneacetonitrile	C6H9NS	-	277	-	31	-	33	80	-	-
**100**	2,6-Nonadienal	C9H14O	-	-	11	-	-	-	17	-	-
**101**	2-Formylhistamine	C6H9N3O	-	-	-	-	33	-	-	-	10
**102**	Hexahydropyrrolizine-3-thione	C7H11NS	-	-	337	197	120	246	148	-	-
**103**	(*R*)-2-(2',2'-Dimethyl-1',3'-dioxolan-4'-yl)ethanal	C7H12O3	-	-	-	-	-	-	16	-	-
**104**	(2*S*,3*R*)-3-Phenyl-2-butanol	C10H14O	-	42	-	-	-	-	-	-	-
**105**	Methoxy phenyl oxime	C8H9NO2	323	499	243	165	224	191	600	640	488
**106**	*trans*-1,1-Dichloro-3,4-epoxypentane	C5H8Cl2O	18	12	15	11	-	-	-	-	-
**107**	Ethyl-3-methylbut-3-enyl carbonate	C8H14O3	-	-	-	-	-	-	25	-	-
**108**	3-Hexyl-1,2,4-trioxolane	C8H16O3	-	-	-	11	-	-	-	-	-
**109**	(2*RS*,4*RS*)-3-*t*-Butylpentane-2,4-diol	C9H20O2	21	-	-	-	-	-	-	-	-
**110**	6,6-Dimethylheptane-2,5-diol	C9H20O2	-	-	-	12	-	-	-	-	-
**111**	(4*R*)-4-Butyl-1,3,2-dioxathiolane-2-oxide	C6H12O3S	-	-	-	-	13	14	64	-	-
**112**	(*R*)-1-(2-Methoxy-6-methyl-phenyl)-ethylamine	C10H15NO	-	-	12	11	-	-	-	-	-
**113**	endo-Bicyclo[3.3.1]non-6-ene-3-carboxylic acid	C10H14O2	-	18	-	-	-	17	-	-	-
**114**	4-Fluorphenoxy-oxivanylmethan	C9H9FO2	71	-	-	-	-	-	-	-	-
**115**	6-Methylhexahydrocycloprop[*a*]pentalen-3a,6-diol	C10H16O2	-	11	-	-	-	-	-	-	-
**116**	Methyl 4,6-decadienyl ether	C11H20O	-	-	-	-	-	-	-	13	-
**117**	*N*-Benzylidene-dimethylammonium chloride	C9H12ClN	-	-	-	69	-	-	260	-	-
**118**	2,2,6,6-Tetramethyl-Cyclohexanmethanol	C11H22O	-	-	-	-	-	-	-	-	13
**119**	2,5-Dimethyl-5-nitrohexanal	C8H15NO3	-	-	-	-	-	-	15	-	-
**120**	5-Mercapto-4-(aminothiocarbonyl)-1-methyl-1,2,3-triazole	C4H6N4S2	-	-	-	-	-	-	12	-	-
**121**	4,4,5-Trimethyl-6,8-dioxa-3-thiabicyclo[3.2.1]octane	C8H14O2S	-	-	-	-	-	33	36	-	-
**122**	3-(2-Methylphenoxy)-3-methoxypropyne	C11H12O2	-	-	27	-	-	-	-	-	-
**123**	1-Mesyloxy-2-methylpent-4-ene	C7H14O3S	-	-	-	-	-	-	-	19	12
**124**	2-Methyl-4-phenyl-3-pentanol	C12H18O	-	12	-	-	-	-	-	-	-
**125**	3-Dodecen-1-al	C12H22O	-	-	-	11	-	11	-	-	-
**126**	Isobutyl 3-methylbut-3-enyl carbonate	C10H18O3	-	-	-	45	-	-	-	-	-
**127**	Isobutyl pent-4-enyl carbonate	C10H18O3	-	-	-	-	-	-	17	-	-
**128**	1,3-Dideoxy-4,5-*O*-(1-methylethylidene)hexitol	C9H18O4	-	-	-	-	-	-	17	-	-
**129**	(*Z*)-2-Hydroxy-5,9-dimethyldeca-4,8-dien-3-one	C12H20O2	-	-	-	11	-	-	-	-	-
**130**	(*S*)-(-)-4-(2-Aminopropyl)imidazole dihydrochloride	C6H13Cl2N3	-	-	-	11	-	-	-	-	-
**131**	2-Phenyl-2-(1,2,3,6-tetrahydro-1-pyridinyl)-1-ethanol	C13H17NO	-	-	-	-	-	-	-	-	25
**132**	(3aS,6R,6aR) 6-Bromohexahydrocyclopenta[*b*]furan-2-one	C7H9BrO2	11	-	-	-	-	-	-	-	-
**133**	γ-Curcumene	C15H24	-	-	-	-	-	-	-	-	30
**134**	6,7-Dimethoxy-2-methyl-3,4-dihydro[1-*D*]isoquinolinium ion	C12H15DNO2	21	291	283	65	749	331	-	-	-
**135**	2-(2,4,4-Trimethylpent-2-yl)phenol	C14H22O	-	-	-	-	-	-	-	-	24
**136**	(*E*/*Z*)-Pentadec-1-yn-3-ene	C15H26	-	11	-	-	-	-	-	-	-
**137**	5-(Phenoxy)methyl-2-amino-1,3,4-thiadiazoles	C9H9N3OS	77	-	46	52	153	-	817	-	-
**138**	2-Nitrophenyl methacrylate	C10H9NO4	-	-	-	-	-	-	10	-	-
**139**	Methyl N-(benzoyl)alaninate	C11H13NO3	11	29	-	-	-	-	-	-	-
**140**	2,4-Nonadienal	C11H13NO3	-	15	-	-	-	13	-	-	-
**141**	Methyl 6-oxo-6(1*H*)-pyrrol-3-yl-2-hexenoate	C11H13NO3	13	-	-	-	-	-	-	-	-
**142**	1-(4-Amino-3-butoxyphenyl)ethanone	C12H17NO2	13	-	-	-	-	-	-	-	-
**143**	1-Benzylindole	C15H13N	-	-	-	-	-	12	11	-	-
**144**	13-Tetradece-11-yn-1-ol	C14H24O	-	-	-	-	-	-	-	-	12
**145**	(3*R*)-3-Phenyl-2,3-dihydro-1*H*-isoindol-1-one	C14H11NO	-	-	-	-	-	-	141	-	-
**146**	E-11,13-Tetradecadien-1-ol	C14H26O	-	-	-	94	-	98	63	-	-
**147**	rac-5-(1-Ethoxyethoy)-3-pentyn-2-yl acetate	C11H18O4	28	-	-	-	-	-	-	-	-
**148**	2,3-Epoxy-1-(methoxymethoxy)nerol	C12H22O3	-	-	-	-	-	-	10	-	-
**149**	Butyl-α-butylacetoacetate	C12H22O3	29	-	-	-	-	-	-	-	-
**150**	5-(4-Methylphenoxy)methyl-2-amino-1,3,4-thiadiazoles	C10H11N3OS	-	-	-	-	78	56	131	-	426
**151**	5-(3-methylphenoxy)methyl-2-amino-1,3,4-thiadiazoles	C10H11N3OS	79	-	47	51	85	-	2359	-	-
**152**	2-Bromo-1-methoxy-4,4-dimethylpentane-3-one	C8H15BrO2	42	-	-	-	-	-	-	-	-
**153**	2-Cyclohexylethyl ethylphosphonofluoridate	C10H20FO2P	-	-	12	35	-	-	-	-	-
**154**	4-Cyclohexyl-5,5-dimethylcyclohexane-1,3-dione	C14H22O2	10	-	-	-	-	-	-	-	-
**155**	*cis*-Sesquisabinene hydrate	C15H26O	-	-	-	-	-	-	-	87	-
**156**	(*Z,E*)-10,12-Pentadecadienol	C15H28O	-	-	-	-	-	-	14	-	-
**157**	Methyl 5-endo-4-hydroxy-2,5,7,7-tetramethylbicyclo[2.2.2]oct-2-en-:	C14H22O3	20	-	-	-	-	-	-	-	-
**158**	(1*R**,4*R**,5*R**)-1,4-Diphenyl-3,6-dioxabicyclo[3.1.0]hexan-2-one	C16H12O3	15	10	-	-	-	-	-	-	-
**159**	2,3,4-Trimethyl-1,3-thiazol-3-ium iodide	C6H10INS	-	399	-	-	-	-	-	-	-
**160**	Di-2,3-methylbutyl maleate	C14H24O4	-	-	-	-	-	-	33	-	-
**161**	Ethyl 3-[(phenacetyl)amino]propane-1-(dithio)-oate	C13H17NOS2	-	25	-	-	-	-	-	-	-
**162**	4-(2-Fluorobenzylidene)-2-phenyl-1,3-oxazol-5(4*H*)-one	C16H10FNO2	77	-	39	44	43	50	79	-	-
**163**	Bis(3,5,5-Trimethylhexyl) ether	C18H38O	-	-	-	-	-	16	-	-	-
**164**	(*S*)-(-)-n-Butyl [(4-chlorophentyl)sulfinyl]acetate	C12H15ClO3S	-	12	-	-	-	13	-	-	-
**165**	2-{Amino[2-cyano-3-(3-nitrophenyl)oxiran-2-yl]-methylidene}malor	C13H7N5O3	129	67	-	81	107	122	-	-	-
**166**	Methyl 2-[4-(1*H*-indol-2-yl)phenoxy]acetate	C17H15NO3	25	-	-	-	-	-	-	-	-
**167**	1,2-Bis(2'-quinolylmethyl)ethylene	C20H14N2	-	31	-	-	-	-	-	-	-
**168**	1-Cyclopentyl-2,3,3,3-tetrafluoro-1-(phenylmethoxy)prop-1-ene	C15H16F4O	-	-	-	-	92	-	-	-	-
**169**	1,1,1-Trifluoro-17-octadecyne	C18H31F3	-	-	-	19	-	16	-	-	-
**170**	1,1,1-Trifluoro-113-octadecyn-2-one	C18H19F3O	-	-	-	11	-	-	-	-	-
**171**	Methyl 3-(1,1'-dicyano-2,2'-bis(rtifluoromethyl)cycloprop-3'-yl)acryl:	C11H6F6N2O2	23	-	-	-	-	-	-	-	-
**172**	2-(2,6-Bis(trifluoromethyl)-9*H*-purin-9-yl)-*N*,*N*-dimethylethanamin	C11H11F6N5	-	-	-	-	-	-	-	-	13

**173**	Limonidilactone	C20H26O4	-	-	-	-	-	-	-	-	15
**174**	3-Formyl-*N*-methyl-9-[phenylethynyl]dibenzo[2,3-a : 5,6-a'] (1,4)-thi	C22H15NOS	-	10	-	12	39	-	13	46	46
**175**	*N*-Benzoyl-5-methyl-3,3-diphenylpyrrolidine	C24H23NO	-	-	-	-	-	-	-	-	13
**176**	Bis(2,6-dimethyl-4-methoxyphenyl)-((1-ethyl)butyl)borane	C24H35BO2	-	-	184	191	87	226	406	-	-
**177**	Ethyl 4-Benzyloxy-2-[2-methyl-2(*E*)-butenyl]-2-[2(*E*),4-pentadienyl	C23H30O4	-	12	-	-	-	-	-	-	-
**178**	1,4-Dipyridylbutane dibromide	C14H18Br2N2	-	-	-	-	-	18	-	-	-
**179**	Phenyl 4-[bis(ethoxycarbonyl)but-3-ynyl]-2,3,4-trideoxy-α, L-glcero-	C21H26O6	16	-	-	-	-	-	-	-	-
**180**	3α-methylcholest-5-en-3-β-ol, nitrite	C28H47NO2	-	-	-	-	13	-	-	16	-
**181**	(*S*)-3,4-Dimethyl-1-pentanol	C7H16O	-	-	-	-	-	-	24	-	-
**182**	3-Methylhexyl isothiocyanate	C8H15NS	-	-	78	62	81	71	32	-	-
**183**	Acorenone B	C15H24O	-	-	-	-	-	-	-	-	16
**184**	1-Isopropyl-1,4,5-trimethylcyclohexane	C12H24	-	-	-	-	-	-	-	-	10
**185**	Nonanenitrile	C9H17N	-	19	-	-	14	-	-	-	-
**186**	1,2-Dipropenyl-cyclobutane	C10H16	-	-	-	-	14	-	-	-	-
**187**	2-Pentadecanone	C15H30O	-	-	-	-	-	-	-	-	19
**188**	2,7-Octanedienol	C8H14O	-	-	-	11	-	-	-	-	-
**189**	2-Octenal	C8H14O	-	-	-	38	31	20	35	24	28
**190**	Pentaethylene glycol monomethyl ether	C11H24O6	-	-	-	-	-	-	11	-	-
**191**	Germacrene D	C15H24	-	-	-	-	-	-	-	-	-
**192**	Acoradiene	C15H24	-	-	-	-	-	-	-	-	57
**193**	2-Undecenal	C11H20O	-	-	19	34	-	43	44	-	-
**194**	1-Methylideneindene	C10H8	-	-	-	-	-	-	-	38	-
**195**	Sulfurous acid, bis(1-methylpropyl) ester	C8H18O3S	-	-	-	-	-	-	-	28	-
**196**	(*E*)-2-Octenal	C8H14O	-	-	12	19	11	-	13	-	-
**197**	(*Z,E*)-α-Farnesene	C15H24	-	-	-	-	-	-	-	-	14
**198**	1*H*,1*H*,2*H*,2*H*-Perfluorodecyl acrylate	C13H7F17O2	29	13	-	-	-	-	-	-	-
**199**	cis-β-Farnesene	C15H24	-	-	-	-	-	-	-	-	84
**200**	6-Methoxy-2-hexanone	C7H14O2	-	-	-	12	18	-	12	-	-
**201**	Cubenene	C15H24	-	-	-	-	-	-	-	53	153
**202**	(*E,E*)-3,5-Octadien-2-one	C8H12O	-	-	22	38	30	25	118	-	-
**203**	4,9-Dipropyldodecane	C18H38	-	-	-	-	-	-	13	-	-
**204**	Pentadecanal	C15H30O	-	-	-	-	-	-	-	51	48
**205**	2-Ethylfuran	C6H8O	-	-	12	18	-	-	19	-	-
**206**	Dodecyloxirane	C14H28O	-	-	-	-	-	-	11	-	-
**207**	3-Butenyl isothiocyanate	C5H7NS	-	14560	26635	20547	14684	23682	12955	11	-
**208**	3-Ethylbenzaldehyde	C9H10O	-	-	69	46	14	42	45	-	-
**209**	3,3,6-Trimethylhepta-1,5-diene	C10H18	-	-	-	13	-	-	-	-	-
**210**	Tricyclo[4.1.0.$0^{2,7}$]hept-3-ene	C7H8	145	91	-	-	-	-	-	-	-
**211**	Dimethyl trisulfide	C2H6S3	-	89	39	43	39	69	25	1417	1070
**212**	3-Ethoxy-3-methylbutan-2-one	C7H14O2	24	-	-	-	-	-	-	-	-
**213**	2-Methyldodecanal	C13H26O	13	-	-	-	-	-	-	-	-
**214**	3-(2-Methylpropyl)cyclopentene	C9H16	-	-	-	-	-	-	11	-	-
**215**	2-Pentylfuran	C9H14O	-	34	-	28	111	-	92	203	155
**216**	2-Benzylbicyclo[2.2.1]heptane	C14H18	-	-	-	-	-	-	-	-	15
**217**	Geranyl acetone	C13H22O	-	-	-	21	-	-	-	64	89
**218**	Thiofanox sulfone	C9H18N2O4S	145	-	-	-	-	-	-	-	-
**219**	4,4-Ethylenedioxy-pentanenitrile	C7H11NO2	-	-	-	-	-	54	-	49	-
**220**	2-Pentadecyl-4,4,5,5-tetradeutero-1,3-dioxolane	C18H32D4O2	-	-	-	685	-	-	-	-	-
**221**	1-Methyl-2-pentyl-Cyclopropane	C9H18	-	-	14	19	-	20	18	-	-
**222**	(*S*)-3-Methyl-1-pentanol	C6H14O	-	-	-	-	-	-	-	-	40
**223**	2-Propylfuran	C7H10O	-	46	-	-	-	-	-	-	-
**224**	Methyl methylthiomethyl disulfide	C3H8S3	-	-	-	-	11	-	-	34	10
**225**	Hexyl Isothiocyanate	C7H13NS	-	-	91	67	40	82	53	-	-
**226**	Heptyl Isothiocyanate	C8H15NS	-	-	52	28	54	52	20	-	-
**227**	4-Ethylcyclohexanol	C8H16O	-	-	-	-	-	-	-	27	26
**228**	3-Pentenenitrile	C5H7N	-	174	-	-	-	-	-	-	-
**229**	α-Cedrene	C15H24	-	-	-	-	-	-	-	62	395
**230**	Ethylcyclobutane	C6H12	-	-	-	-	-	-	19	20	25
**231**	Cadina-1,3,5-triene	C15H22	-	-	-	-	-	-	-	-	87
**232**	Zingiberene	C15H24	-	-	-	-	-	-	-	13	41
**233**	β-Bisabolene	C15H24	-	-	-	-	-	-	-	-	41
**234**	5-Cyano-1-pentene	C6H9N	-	1079	390	467	961	296	348	609	468
**235**	3-Methyl-4-penten-1-ol	C6H12O	-	-	37	-	-	-	-	-	-
**236**	Acetoin	C4H8O2	257	-	-	-	-	-	-	4328	4124
**237**	4-Ethyl-5-methylthiazole	C6H9NS	-	9728	28848	24592	17509	26974	21369	61	90
**238**	Hydroxyethyl methyl sulfide	C3H8OS	-	-	-	-	-	-	-	-	13
**239**	1-Methoxy-2-butanol	C5H12O2	-	-	-	-	-	-	-	10	-
**240**	Citral	C10H16O	-	-	-	-	-	-	30	97	37
**241**	4,5-Epithiovaleronitrile	C5H7NS	-	422	50	-	23	50	51	-	-
**242**	Pentanedinitrile	C5H6N2	-	-	355	-	754	-	234	-	530
**243**	Nonyl chloroacetate	C11H21ClO2	-	-	-	-	-	-	-	24	22
**244**	1-Methoxyindole	C9H9NO	-	-	-	-	51	-	62	-	-
**245**	1,2-dimethyl 1,2,4-benzenetricarboxylate	C11H10O6	-	-	-	-	-	-	-	-	77
**246**	cis-5-Methyl-cis-1,3-cyclohexanediol	C7H14O2	-	64	-	-	-	-	-	-	-
**247**	Benzylhydrazine	C7H10N2	-	-	-	-	20	-	-	-	-
**248**	Methyl sulfocyanate	C2H3NS	-	-	-	-	-	-	221	381	148
**249**	(*E,Z*)-2,6-Nonadienal	C9H14O	-	-	-	15	-	-	-	-	-
**250**	4-Pentenyl pentyl ether	C10H20O	-	-	-	-	-	11	-	-	-
**251**	2,3-Dihydro-3-(1-methylpropyl)furan	C8H14O	-	-	-	-	-	12	-	-	-
**252**	5,6-dimethylidenebicyclo[2.2.1]hept-2-en-7-one	C9H8O	-	24	-	11	-	16	-	-	-
**253**	(*E*)-1,3,7-octatrien-5-yne	C8H8	-	-	-	-	-	-	15	-	-
**254**	4,7,10,13,16-Pentaoxanonadeca-1,18-diene	C14H26O5	-	-	40	26	23	27	21	38	50
**255**	2,5-Dichlorophenol	C6H4Cl2O	-	-	-	-	-	-	-	-	40
**256**	2,2-Diacetylindan	C13H14O2	-	-	-	-	-	-	-	-	10
**257**	(*Z,E*)-2,4-Nonadienal	C9H14O	-	26	18	18	39	28	37	-	-
**258**	2-Hexanone	C6H12O	30	-	-	-	-	-	-	-	-
**259**	1-Ethylpentyl acetate	C9H18O2	-	15	-	-	-	-	-	-	-
**260**	1-Isothiocyanatobutane	C5H9NS	-	71	29	29	-	30	35	-	-
**261**	2-Tridecanone	C13H26O	-	-	-	-	-	-	-	23	71

**262**	4,4-Dimethylhexanal	C8H16O	-	-	-	-	-	-	-	12	-
**263**	2,3-Pentanedione	C5H8O2	-	-	-	-	-	-	-	-	20
**264**	cis-1,2-Diethylcyclobutan	C8H16	-	-	-	-	-	-	48	-	-
**265**	4,4-Dimethyl-5-oxopentanenitrile	C7H11NO	-	-	-	-	10	-	-	-	-
**266**	Benzyl isothiocyanate	C8H7NS	-	-	-	-	-	-	16	-	-
**267**	Dimethyl disulfide	C2H6S2	-	147	68	91	79	190	549	3203	1862
**268**	2-Methyl-2-octanol	C9H20O	18	-	-	-	-	-	-	-	-
**269**	Heptanonitrile	C7H13N	-	16	-	-	-	-	-	-	-
**270**	Tetradecane	C14H30	-	-	-	-	-	-	-	15	-
**271**	Phenylsuccinic acid	C10H10O4	-	25	-	-	-	-	-	-	-
**272**	(*Z*)-5-Octenol	C8H16O	-	-	-	-	-	-	-	16	13
**273**	1-(1,5-Dimethyl-4-hexenyl)-4-methylbenzene	C15H22	-	-	-	-	-	-	-	49	305
**274**	Benzenepropanenitrile	C9H9N	-	1851	752	664	482	720	514	484	354
**275**	6-Methoxyquinoline 1-oxide	C10H9NO2	-	-	-	-	-	-	-	-	53
**276**	(2,2-dimethyl-3-phenyl-azetidin-1-yl)-oxo-acetonitrile	C13H14N2O	-	-	-	-	-	15	-	-	14
**277**	1,1,2,2-Tetrahydroperfluoro-1-decanol	C10H5F17O	23	-	-	-	-	-	-	-	-
**278**	2,2,4-Trimethyl-1,3-pentanediol diisobutyrate	C16H30O4	-	-	-	28	24	31	32	-	-
**279**	2-Ethylhexyl methacrylate	C12H22O2	-	-	-	41	-	-	-	-	-
**280**	4-(1,1-Dimethylethyl)-2-[(1-methylethoxy)methylene]-cyclohexanon	C14H24O2	-	-	-	-	-	-	-	-	16
**281**	4-Methylisothiazole	C4H5NS	-	-	20	20	-	21	-	-	-
**282**	(*R,R*)-2,3-Butanediol	C4H10O2	-	-	-	-	-	-	-	26	-
**283**	cis-2-(2-Pentenyl)furan	C9H12O	-	-	11	13	-	-	28	-	-
**284**	2-methoxy-[1]Benzothieno[2,3-c]quinolin-6(5H)-one	C16H11NO2S	72	36	34	34	36	30	219	-	-
**285**	2,3-Dimethyloctane	C10H22	-	-	-	-	-	-	39	-	-
**286**	(1-Hydroxy-2,4,4-trimethylpentan-3-yl) 2-methylpropanoate	C12H24O3	14	-	-	-	-	-	-	-	-
**287**	2,6-Dimethyltetraline	C12H16	-	-	-	-	-	-	-	-	13
**288**	Isoamyl pyruvate	C8H14O3	-	-	-	-	-	-	-	142	90
**289**	2-Methyl-2,3-pentanediol	C6H14O2	13	-	-	-	-	-	-	22	-
**290**	Methyl (2*E*)-5-(1-ethoxyethoxy)-2-hexenoate	C11H20O4	-	359	39	-	-	-	-	-	-
**291**	4,4,6-Trimethyl-1,3-thiazinane-2-thione	C7H13NS2	-	-	158	-	181	318	-	-	-
**292**	(*Z*)-3-Decenyl acetate	C12H22O2	-	-	-	-	-	-	-	-	15
**293**	4-Pentenol	C5H10O	-	-	-	-	-	-	-	-	12
**294**	2-Nonanone	C9H18O	-	-	-	-	-	-	-	46	68
**295**	[3-(2-methyl-1-oxopropyl)oxiranyl] phosphonic acid diethyl ester	C10H19O5P	-	-	-	-	-	-	-	-	18
**296**	13-Tetradecenal	C14H26O	-	-	-	-	-	-	-	60	61
**297**	Nitrosomethane	CH3NO	-	-	-	-	-	-	-	4889	1609
**298**	*S,S*-Dimethyl dithiocarbonate	C3H6OS2	-	-	-	-	-	-	-	34	-
**299**	(*E,E*)-2,4-Heptadienal	C7H10O	-	99	252	302	169	181	385	46	-
**300**	4-Hydroxy-2(1H)-Pyridinone-5,6-d2	C5H3D2NO2	-	-	-	-	-	-	-	42	61
**301**	6-methyl-6-(3'-isopropenyl-2'-methyl-cycloprop-1'-en-1'-yl)-2-heptan	C15H26O	-	-	-	-	-	-	-	-	12
**302**	2,3-Epoxy-3-methyl-1-phenylbutane	C11H14O	11	-	-	-	-	-	-	-	-
**303**	Cyclobutanecarbonitrile	C5H7N	-	193	96	99	151	70	47	-	-
**304**	(*E*)-4-Hexen-1-ol	C6H12O	-	-	-	-	-	-	-	14	-
**305**	(*E*)-3-Hexen-1-ol	C6H12O	-	-	-	46	-	-	54	-	-
**306**	Decyl cyclohexanecarboxylate	C17H32O2	-	-	56	-	-	-	-	-	-
**307**	Bis(2,6-dimethyl-4-methoxyphenyl)-((1-ethyl)butyl)borane	C24H35BO2	307	-	-	-	-	-	-	-	454
**308**	1-Phenylpropane-1,2-diol	C9H12O2	-	-	-	-	-	-	-	104	159

Data are shown as the mean (n=3).

- : Not detectable.
